# Cholagogues

**Published:** 1878-10-20

**Authors:** 


### CHOLAGOGUES.

Professor Rutherford pointed out that clinical observation was very defective in regard to the action of medicines on the liver. It was difficult to ascertain the amount of bile secreted. Some substances resembled the bile in color, as rhubarb. Again, when bile had been secreted, it was sometimes retained in the alimentary canal, especially if the cholagogue were not at the same time a purgative, as was the case with many substances which acted on the liver. Again it had been proved by his experiments that all medicines which acted as purgatives, but did not stimulate the liver, lessoened the secretion of bile. This law had been clearly demonstrated. After reviewing the methods of experimenting upon the liver by former experimenters, as Hughes Bennett, Rohrig, and others, and pointing out wherein their methods were defective or fallacious, Professor Rutherford briefly des-

*Paper presented to the Medico-Chirurgical Society of Edinburgh, June 19th, 1878.

cribed how his experiments had been conducted. They were all performed upon dogs in a state of fasting. The animal was fed at 4 P. M., and the experiment was commenced about 9 o'clock the following morning. The animal was kept perfectly still by means of curara, which paralyzed its movements, and artificial respiration was kept up by making an opening into the trachea and introducing a pair of bellows, worked by a small steam engine. The respirations at sixteen per minute. It was necessary to keep the dog in a state of fasting, for when food was being absorbed the hepatic cells were rendered more active. It was necessary to use curara, for chloroform and ether both increased the activity of the liver. Several observations were first made to ascertain how much bile was secreted normally by the liver in a given time. The amount was measured every quarter of an hour, and the experiment lasted nine or ten hours. An opening was made into the abdomen in the linea alba, a canula was introduced into the common bile duct, and the bile was allowed to fall into a fine cubic centimetre measure. The gall-bladder was squeezed so as completely to fill the canula, and a clamp was placed on the cystic duct to prevent the return of the bile into the gall-bladder. It was found that the flow of bile when no substance was applied was tolerably regular, but had a tendency to diminish after several hours, owing to the exhaustion of the liver. The substances were all introduced into the duodenum for two reasons; first, on account of the large amount of mucin in the stomach of the dog; and secondly, because many maintained that the action of cholagogues was due to their irritating the mouth of the common bile duct.

Professor Rutherford experimented with the following substances:

1. Croton Oil.—He commenced with this substance because, according to Rohrig, it was the most powerful cholagogue. In one experiment he gave 15 grains, in another 3 grains of croton oil, and he found from these experiments that croton oil was a cholagogue of such feeble nature that it might well be discarded. He tried also the action of water alone, and found that it did not affect the secretion of bile to any notable extent.

2. Podophylline.—He injected 10 grains of this substance, and found only a very slight increase of bile, because it acted as a too powerful purgative. In another case he gave 6 grains of podophlline, and there was a very constant rise in the secretion of bile. As resinoid substances were very insoluble unless mixed with bile, which dissolved such substances, he injected 9 grains of podophylline with a little bile, having previously ascertained that such an addition did not notably affect the secretion of bile. In this case there was much purgation, and for the first half hour the secretion of bile rose enormously, but afterwards fell almost to a stand still. From these experiments he found that podophylline was a powerful cholagogue. As the experiment occupied a great deal of time Professor Rutherford associated M. Vignal with him in their execution.

3. Castor Oil.—This was the next substance tried. He gave one ounce and found that it did affect the liver. On the other hand, as purgation set in there was a diminutión of the secretion of bile due to the purgation.

4. Gamboge.—He gave 4 grains, and there was no rise, but a decided fall, due to excessive purgation.

5. Sulphate of Magnesium.—He gave 60 grains, and after a time another 60 grains, but noticed only a fall in the quantity of bile secreted.

5. Chloride of Ammonium.—He gave about 20 grains of this substance four times repeated, and the secretion fell. The effect of purgation was to diminish the bile by draining the portal system. Part of the bile was normally reabsorbed, and especially the sulphur-containing substance, and reconverted into taurocholic acid; but purgatives prevented this by washing away the bile after it had been poured into the alimentary canal.

7. Aloes.—He gave 60 grains, and the secretion of bile rose high, and remained so for a long time. Small doses affected the secretion of bile only slightly.

8. Rhubarb.—He gave 17 grains four times in the form of infusion, and the secretion of bile rose afterwards. Rhubarb did increase the secretion of bile, but was a feeble hepatic stimulant.

9. Senna.—He gave 45 grains five times. It stimulated the liver, but was not a very powerful cholagogue, being accompanied by purgation.

10. Colchicum.—He gave 60 grains of the extract, and there was a very high secretion. It was a decided cholagogue, but not a powerful one.

11. Taraxacum.—He gave 180 grains of the extract and afterwards I20 grains, and the secretion of bile rose, but so slightly that he concluded that taraxacum was an extremely feeble cholagogue.

12. Scammony.—He gave 4 grains and afterwards 8 grains, and found it a very feeble cholagogue.

